# Shadow of domestic violence and extramarital sex cohesive with spousal communication among males in Nepal

**DOI:** 10.1186/1742-4755-11-44

**Published:** 2014-06-13

**Authors:** Dharma Nand Bhatta

**Affiliations:** 1 Department of Public Health, Pokhara University, Nobel College, Sinamangal, Kathmandu, Nepal

**Keywords:** *Violence*, *extramarital sex*, *Contraceptive*, *Spousal communication*, *Nepal*

## Abstract

**Background:**

Public health and human right issues are challenging in low and middle income countries. The main objectives of this paper were to determine the prevalence and factors associated with domestic violence, extramarital sex, and spousal communication among male.

**Methods:**

A cross-sectional study among 2466 married males in Kathmandu, Nepal was conducted using random sampling method. Adjusted odds ratios (AORs) and 95% confidence intervals (CI) of associated factors were estimated by stepwise backward likelihood ratio method.

**Results:**

Prevalence of domestic violence was 63.14% (95% CI 61.20-65.05), extramarital sex was 32.12% (95% CI 30.27-34.00), and spousal communication was 48.87% (95% CI 46.85-50.90). Nearly one in five male (18.20%) had not used condom during extramarital sex.

Interestingly, male who had more than three or equal children were less likely to have perpetrated domestic violence compared with those who had less children. Older male aged 25 and above were more likely (AORs = 1.55, 95% CI 1.19-2.03) to have extramarital sex compared with male aged 24 or below. Those male who had studied secondary or higher level of education were less likely to have extramarital sex compared to those who had primary level or no education. Male who had higher income were more likely to have spousal communication compared to those who had less income. Surprisingly, those male who had extramarital sex were less likely to have spousal communication compared with those was not involved in extramarital sex.

**Conclusion:**

Practice of domestic violence and extramarital sex is quite common among married male in Nepal, where spousal communication is sparse. These findings can be used to advocate for immediate attention and activities needs to be endorsed by policymakers and programmers.

## Background

Globally violence against women is a grave fact which hacks every social and economic class families. According to World Health Organization (WHO), 10-69% of women experienced with physically beaten by husband at several point in their lives [1]. Psychological and sexual violence is greatly common in the world, which has great public health impacts [1-3], viz. HIV, alcohol abuse and drug addiction, homicide and suicides, mental disorders, miscarriages, preterm labor, fetal distress and low birth weight infants [4-6]. Globally more than fifty population based studies revealed that around 10-50% of women reported having been physically assaulted by male partner [7]. Similarly, study conducted by WHO reported that 13-61% of women had experienced physical violence and 10-50% of women informed being sexually abused [8]. Study from India showed that 24% being kicked, 12% were threatened by their husbands [9]. Study from Bangladesh revealed that 67% of women accounted experiencing domestic violence [6]. Likewise, a study conducted in Nepal reported 46.2% of women reported experiencing sexual violence [10]. Albeit the degree of indication has been mounting worldwide and studies in Nepal to assess the prevalence of violence in different settings and populations is sparse. A higher percentage of married accounted experiencing violence contrasted to unmarried [11]. WHO reported that unnatural sex by male partners ranged from 4% in Serbia and Montenegro to 46% in Bangladesh and Ethiopia [12].

Domestic violence has strong association with low income, low education level of husband, alcohol and drug use, young age, and eye witnessing DV during childhood, HIV status, polygamy, multiple sexual partners, use of contraceptives, woman’s power over resources, woman’s education, employment and culture [13-24]. Intimate partner violence is strongly connected with male patriarchal principles [25]. Patriarchal family, culture, society and religious principles in Nepal enforce the violence [26]. Though, government of Nepal passed a law on gender-based violence which made punishment range from a fine or six months in jail or both depending on the type of violence, however this law is seldom imposed strictly [27,28].

Both married men and women have reported the practice of extramarital sexual and more frequent among men. Study from Nigeria revealed that the 79% of transport workers, 46% of army, 61% of policeman had engaged in extramarital sex and demographic and health survey showed that 11% of married men reported having had extramarital sex [29,30]. Men with more than one wives are more likely to have extramarital sex and always at risk of extramarital sex [30]. It has been established in many countries that HIV is transmitted typically throughout sexual behaviors [31]. Previous studies from other countries have highlighted the risk of men infecting their wives after extramarital sex during periods of separation and the continuing impact of the epidemic is expected to be determined by men who have extramarital sex [32,33].

Fertility indicators have been influenced by changes in marital relationships [34,35]. Spousal communication has concern primarily husbands’ and wives’ discrepancy information of communication on fertility and family planning [36-38]. Limitations of the various studies cannot make causal inferences whether communication leads contraceptive use or not [39,40]. Various factors play an important role in husband wife relationship including vivid social, economic and institutional changes and cultural shift [41]. Spousal communication is the vital factor which helps to change the attitude and intention of the people in the use of contraceptives. Studies from Africa observe immense agreement highlighting on spousal communication and contraceptive use [36,39]. Those researches have repeatedly recommended that women play the crucial role in making contraceptive choices [42]. Ignored role of men’s in contraceptive manners might be warning the achievement of family planning (FP) [40].

The main objective is to determine the prevalence and factors associated with domestic violence, extramarital sex and spousal communication. Culturally vibrant and patriarchic societies of Nepal with low socio-economic, decision-making abilities of women and beliefs have immense impact on women’s health [43]. Urgent needs to address these factors which help to improve women’s health.

## Materials and methods

### Design and settings

A cross-sectional study was carried out in different twenty three village development committees (VDCs) and one municipality of Kathmandu district which is the central part of Nepal. Study was conducted from May to December 2010. A married male of a household head and interest to participate in the study were the inclusion criteria.

### Sampling and sample

Two-staged random sampling technique was applied in order to sample the married males. The first stage of sampling included random selection of 23 VDCs and one municipality in Kathmandu. In order to select these 23 VDCs and one municipality, a list of all the VDCs and municipalities those located in Kathmandu was obtained from the office of the District Development Committee in Kathmandu. This list included all the wards of VDCs and municipalities. In the second stage, one ward was selected randomly from each sampled VDCs and municipality. Households from these wards were not differentiated by subject and had taken in the same geographical area. The number of married males of household heads in each wards ranged from 200 to 400 households. To reach sampled household heads in 23 wards of VDCs and one ward of municipality, 100 household heads from each VDC and 200 household heads from municipality were taken for interview as a sample. Among the total 2500 household heads, 34 were refused to take participation in the study. Hence the total sample size was estimated to be 2466 male.

### Data collection and measures

Structured questionnaires were used for the data collection and included questions on socio-demographic characteristics of the respondent, along with his practice of domestic violence, extramarital sex and spousal communication. Pre-testing of the questionnaires with married male in non-sampled areas was carried out and the necessary changes in the questionnaire were incorporated.

The questionnaires were drafted in English and translated into Nepali, Nepal’s most common language. All the changes were methodically checked to ensure that meaning of the original English version was maintained. Researchers were trained with regards to ethical thoughts while conducting research on sensitive issues [43]. Participants involved in the study were fully informed about the nature of the study, objectives, and confidentiality of the data. Each participant’s verbal consent was obtained after assuring confidentiality. The face-to-face interview was conducted in a secret place without the presence of third person. The researcher did not give any financial recompense to the interviewed male. Confidentiality of information was guaranteed by removing individual identifiers from the fulfilled questionnaires, which sheltered respondents against unpleasant impacts from participating in the study. The institutional ethical review committee has approved this study.

Domestic violence perpetrated by a male is used as the dependent variable in this study. Respondents were asked three questions:

i. Have you ever been practiced any one of these activities (slapped, or pushed, or kicked, or beaten, or choked) with your wife?

ii. Have you ever been forced your wife to have sexual intercourse?

iii. Have you ever been embarrassed your wife?

Those men who performed at least one of the three forms of violence were considered to have performed domestic violence by male. Domestic violence, extramarital sex and spousal communication also measured as a primary outcomes or dependent variable. With regard to extramarital sex, this measure was focused on whether they had had sex even once before ever with other than wife. Similarly, the question on extramarital sex was dichotomized into yes and no. Likewise, spousal communication variable was based on the history of discussion with their wife regarding fertility, and FP which was then dichotomized into yes and no. Rest of the questions related on contraceptive use was also coded as yes and no. Contraceptive used by males and male decision maker for contraceptive uses were considered as secondary measures.

### Data analysis

The coded data was entered into SPSS version 16 for further processing and analysis. Bivariate and multivariate techniques were applied to identify the factors associated with the likelihood of practicing domestic violence, extramarital sex, and spousal communication. Researcher used the Hosmer and Lemeshow test to assess goodness of fit of the models and the likelihood ratio test to assess relative contribution of terms entered into the model. The variables were also examined in the multivariate analysis in order to identify the significant predictors after controlling for other variables. Logistic regression results were presented as both crude and adjusted odds ratios (AORs) with 95% confidence intervals. In the bivariate and multivariate analyses, p-value of less than or equal to 0.05 was considered to be significant. The data was cleaned and cross-checked daily before and after data entry for completeness and accuracy.

## Results

The respondents mean age ± SD was 32.4 ± (8.9) years, where a large majority of the respondents (81.38%, 95% CI 79.80-82.90) were aged 25 years and above. Out of total respondents, 72.26% (95% CI 70.45-74.02) of the male had secondary or higher level education, 48.91% (95% CI 46.91-50.90) of the male had formal employment, 88.32% (95% CI 86.99-89.56) of the male followed Hindu religion, and 82.48% (95% CI 80.92-83.96) of the male were from non-indigenous ethnicities. Majority of the respondents (67.77%, 95% CI 66.76-69.73) had less than or equal two children, whereas 16.67% (95% CI 15.21-18.20) of the male had desire for more children (Table 1).

**Table 1:** Socio-demographic information (N = 2466)

**Characteristic**	**Frequencies (Percentage)**	**95% CI**
**Age	32.4 ± (8.9)	-
25 years and above	2007 (81.38)	79.80 - 82.90
24 years and young	459 (18.62)	17.09 - 20.21
Income level Monthly (NPR)*		
5001.00 NPR or above	1188 (48.17)	46.18 – 50.16
5000.00 NPR or below	1278 (51.83)	49.83 – 53.81
**Education level**		
Secondary or Higher Level	1782 (72.26)	70.45 – 74.02
Uneducated or Primary Level	684 (27.74)	25.97 – 29.55
**Occupation**		
Formal employment	1206 (48.91)	46.91 – 50.90
Informal or no employment	1260 (51.09)	49.09 – 53.09
**Religion**		
Hindu	2178 (88.32)	86.99. – 89.56
Non-Hindu	288 (11.68)	10.44 – 13.01
**Ethnicity**		
Non-Indigenous	2034 (82.48)	80.92 – 83.96
Indigenous	432 (17.52)	16.04 – 19.08
**No. of Children (N = 2178)	2.17 ± (1.09)	-
≤ 2 Children	1476 (67.77)	66.76 – 69.73
≥ 3 Children	702 (32.23)	30.27 – 34.24
Desire more children	441 (16.67)	15.21 – 18.20

*1 U.S. $ = 85.00 NPR **Mean ± standard deviation CI = Confidence Interval.

A large majority of the respondent (81.82%) had used condom during extramarital sex. Among the 1710 respondents, male said that the reasons for not to use FP method were: the feeling that this is a woman’s duty (15.81%), it reduced sexual power (23.20%), it reduced working power (32.12%), had cultural and religious barriers (15.31%), and had other causes (13.71%) (Table not shown).

Table 2 shows the indication of male influences in different issues. Regarding decision making for family planning, two third of the respondent (66.16%, 95% CI 64.23-68.06) indicated that they were decision makers for FP. Large majority of the wives (92.10%, 95% CI 90.95-93.15) needed approval or consent to use contraceptives. This is in spite of the claimed by most of the respondents that they discussed about family planning issues. Overall, half of the male (48.87%, 95% CI 46.85-50.90) indicated that they had spousal communication regarding fertility, and FP. Overall, nearly two third of the male (63.14%, 95% CI 61.20-65.05) reported that they had perpetrated domestic violence with their wives. One third of the respondents (32.12%, 95% CI 30.27-34.00) reported that they had extramarital sexual relation. Underreporting of such sexual activities was highly possible due to the sensitive nature of the study.

**Table 2:** Male influence on different issues (N = 2394)

**Characteristic**	**Frequencies (Percentage)**	**95% CI**
Family planning method used by men	684 (28.57)	26.76 – 30.43
Men decision maker for contraceptive use	1584 (66.16)	64.23 – 68.06
Wife decision maker to use contraceptive	90 (03.76)	03.03 – 04.60
Wife needed approval to use contraceptive	2205 (92.10)	90.95 – 93.15
Spousal communication regarding family planning	1170 (48.87)	46.85 – 50.90
Family planning clinic visit with wives	261 (10.90)	09.68 – 12.22
Discussion with wives regarding HIV and AIDS (N = 2466)	972 (39.42)	37.47 – 41.38
Extramarital sexual relation	792 (32.12)	30.27 – 34.00
Men attempt domestic violence	1557 (63.14)	61.20 – 65.05

Those male who had more than three or equal children were less likely to have perpetrated domestic violence compared with those who had less than two or equal children. Male aged 25 and above (AORs = 1.59, 95% CI 1.18-2.16), had education level secondary or higher (AORs = 1.68, 95% CI 1.35-2.09), had income 5001 NPR or above per month (AORs = 1.78, 95% CI 1.42-2.21), had formal employment (AORs = 1.68, 95% CI 1.34-2.09) and were non-indigenous ethnicities (AORs = 1.70, 95% CI 1.30-2.23) have more likely to have perpetrated domestic violence (Table 3).

**Table 3:** Associated factors for domestic violence (N = 2466)

**Characteristic**	**Crude odds ratios with 95% confidence interval**	**Adjusted odds ratios with 95% confidence interval**
Age above 25 years	1.09 (.88 – 1.34)	1.59 (1.18 – 2.16)** ^d^
Age 24 years & below -Ref		
Education secondary or higher	2.33 (1.90 - 2.84)*	1.68 (1.35 - 2.09)* ^c^
Uneducated or primary level- Ref		
Income 5001 NPR or above	2.19 (1.85- 2.59)*	1.78 (1.42-2.21)* ^d^
5000 NPR or less/month- Ref		
Formal employment	2.23 (1.89 - 2.64)*	1.68 (1.34 - 2.09)* ^d^
Informal or no employment - Ref		
Non-Indigenous ethnicity	2.23 (1.75 - 2.83)*	1.70 (1.30 - 2.23)* ^d^
Indigenous ethnicity - Ref		
≥ 3 Children	.54 (.44 - .67)*	.60 (.48 - .75)* ^d^
≤ 2 Children - Ref		

*P < 0.001, **P < 0.05.

^c^Adjusted with education, age, ethnicity, no. of children.

^d^Adjusted with age, income, employment, ethnicity, no. of children.

Findings regarding extramarital sexual relation revealed that the older male aged 25 and above (AORs = 1.55, 95% CI 1.19-2.03), had income 5001 NPR (1 USD = 90 NPR) or above per month (AORs = 1.24, 95% CI 1.02-1.49), and had more than three or equal children (AORs = 1.97, 95% CI 1.59-2.45) were more likely to have extramarital sex compared with male aged 24 or below, had income 5000 NPR or below, and had less than two or equal children. However, those male who had studied secondary or higher level of education and had desire for more children were less likely to have extramarital sex compared with those who had primary level or no education and had no more desire for children (Table 4).

**Table 4:** Associated factors for extramarital sex (N = 2466)

**Characteristic**	**Crude odds ratios with 95% confidence interval**	**Adjusted odds ratios with 95% confidence interval**
Age above 25 years	1.99 (1.62 - 2.46)*	1.55 (1.19-2.03)*^a^
Age 24 years & below -Ref		
Education secondary or higher	.62 (.51 - .76)*	.77 (.62 - .95)**^a^
Uneducated or primary level- Ref		
Desire more children	.29 (.24 - .36)*	.34 (.27 - .43)*^a^
No more desire - Ref		
Income 5001 NPR or above	1.26 (1.06-1.49)**	1.24 (1.02-1.49)**^b^
5000 NPR or less/month- Ref		
Non-Indigenous ethnicity	1.49 (1.20-1.85)*	1.42 (1.13-1.78)**^b^
Indigenous ethnicity - Ref		
≥ 3 Children	1.91 (1.55-2.35)*	1.97 (1.59-2.45)*^b^
≤ 2 Children - Ref		

*P < 0.001, **P < 0.05.

^a^Adjusted with age, education, desire of children, income, ethnicity, no. of children.

^b^Adjusted with income, ethnicity, no. of children.

Table 5 indicates the factors associated with spousal communication. The analysis found that male who had desire for more children were three times (AORs = 3.00, 95% CI 2.32-3.90) more likely to have spousal communication compared to those who had no more desire for children. Male with more than three or equal children (AORs = 1.30, 95% CI 1.04-1.61), and income 5001 NPR or above per month (AORs = 1.22, 95% CI 0.98-1.52) were more likely to have spousal communication compared to those who had less than two or equal children, and had income 5000 NPR or below. Unexpectedly, those male who had studied secondary or higher level of education, and had extramarital sex were less likely to have spousal communication compared with those who had primary level or no education and were not involved in extramarital sex.

**Table 5:** Associated factor for spousal communication (N = 2394)

**Characteristic**	**Crude odds ratios with 95% confidence interval**	**Adjusted odds ratios with 95% confidence interval**
Income 5001 NPR or above	.60 (.51- .70)*	1.22 (.98-1.52)^c^
5000 NPR or less/month- Ref		
Education secondary or higher	.28 (.23- .34)*	.33 (.26-.41)*^c^
Uneducated or Primary level- Ref		
Formal employment	.36 (.31-.43)*	.65 (.52-.82)*^c^
Informal or no employment - Ref		
Hindu religion	.27 (.20- .36)*	.50 (.35-.72)*^c^
Non-Hindu - Ref		
Non-Indigenous ethnicity	.35 (.27-.44)*	.75 (.56-1.02)^c^
Indigenous ethnicity - Ref		
≥ 3 Children	1.32 (1.09- 1.59)**	1.30 (1.04- 1.61)**^c^
≤ 2 Children - Ref		
Desire more children	2.67 (2.13- 3.34)*	3.00 (2.32- 3.90)*^c^
No more desire - Ref		
Extramarital sexual relation	1.00 (.84- 1.19)	.79 (.64- .97)**^c^
No relation - Ref		
**Associated factors for wife’s approval to use contraceptive (N = 2394)**		
Age above 25 years	2.37 (1.44-3.90)*	4.47 (2.21-9.03)*^a^
Age 24 years & below -Ref		
Income 5001 NPR or above	1.72 (1.27 - 2.33)*	1.52 (1.12-2.07)**^b^
5000 NPR or less/month- Ref		
Education secondary or higher	7.53 (3.83-14.80)*	5.14 (2.57 -10.27)*^a^
Uneducated or Primary level- Ref		
Hindu religion	2.79 (1.41-5.52)**	2.5 (1.12-5.71)**^a^
Non-Hindu - Ref		
Non-Indigenous ethnicity	1.28 (.84-1.95)	.51 (.30- .87)**^a^
Indigenous ethnicity - Ref		
≥ 3 Children	.13 (.07-.26)*	.14 (.07- .27)*^a^
≤ 2 Children - Ref		
Desire more children	.80 (.52-1.22)	.67 (.42-1.06)^a^
No more desire - Ref		

*P < 0.001, **P < 0.05.

^b^Adjusted with age, income, religion.

^a^Adjusted with age, education, religion, desire of children, ethnicity, no. of children.

^c^Adjusted with income, education, employment, religion, ethnicity, desire of children, extra marital sexual relation, no. of children.

Those male who had studied secondary or higher level of education were five times (AORs = 5.14, 95% CI 2.57-10.27) more likely to have their wives needed approval to use contraceptive compared with those who had primary level or no education. Those male who believed in Hindu religion were about three times (AORs =2.50, 95% CI 1.12-5.71) more likely to have their wives needed approval to use contraceptive compared to those who believed in other than Hindu religion. However, those male who had more than three or equal children and had desire for more children were less likely to have their wives needed approval to use contraceptive compared with those who had less than two or equal children and had no more desire for children.

Table 6 shows the factors associated with FP methods used by men. Education, age, income, desire for more children, attempted domestic violence and the number of surviving children were found to have a significant impact on contraceptive use. Furthermore, the variables age, level of education, religion and ethnicity had statistical significant effect on decision making for contraceptive use. Unexpectedly, those male who had studied secondary or higher level of education were four times (AORs = 4.11, 95% CI 3.12-5.42) more likely to have decision makers for contraceptive use compared with those who had primary level or no education. Interestingly, those male who had attempted domestic violence were less likely to have decision makers compared with those who did not have such activities.

**Table 6:** Associated factors for FP method use (N = 2394)

**Characteristic**	**Crude odds ratios with 95% confidence interval**	**Adjusted odds ratios with 95% confidence interval**
Age above 25 years	1.65 (1.33-2.04)*	.74 (.54-1.02)
Age 24 years & below -Ref		
Income 5001 NPR or above	.85 (.71-1.00)	1.30 (1.04-1.62)**
5000 NPR or less/month- Ref		
Education secondary or higher	.35 (.27-.44)*	.41 (.31-.55)*
Uneducated or Primary level- Ref		
Hindu religion	.15 (.09-.24)*	.09 (.05-.18)*
Non-Hindu - Ref		
≥ 3 Children	2.48 (1.93-3.19)*	2.05 (1.56-2.71)*
≤ 2 Children - Ref		
Desire more children	.78 (.62-.99)**	.72 (.55-.95)**
No more desire - Ref		
Men attempt domestic violence	1.87 (1.56-2.24)*	1.29 (1.04-1.61)**
Not attempt - Ref		
**Associated factors for male decision making to contraceptive use (N = 2394)**		
Age above 25 years	1.63 (1.30-2.05)*	1.57 (1.15-2.14)**
Age 24 years & below -Ref		
Income 5001 NPR or above	1.09 (.92-1.30)	.67 (.54-.82)*
5000 NPR or less/month- Ref		
Education secondary or higher	4.54 (3.53-5.84)*	4.11 (3.12 - 5.42)*
Uneducated or Primary level- Ref		
Hindu religion	2.32 (1.70-3.16)*	1.43 (.95-2.16)
Non-Hindu - Ref		
Non-Indigenous ethnicity	2.40 (1.85 -3.10)*	1.49 (1.06-2.10)**
Indigenous ethnicity - Ref		
≥ 3 Children	.67 (.54-.82)*	.70 (.55-.88)**
≤ 2 Children - Ref		
Desire more children	.64 (.50-.82)*	.66 (.50-.87)**
No more desire - Ref		
Men attempt domestic violence	.62 (.52-.73)*	.83 (.68-1.02)
Not attempt - Ref		

*P < 0.001, **P < 0.05.

## Discussion

The results of the study indicated that prevalence of domestic violence is high. The finding is similar with previous studies from different countries [1,7-12]. Extramarital sex is unacceptable in Nepalese society and the results indicated that one third of the men have extramarital sexual relations. A study from Cameroon has revealed similar result [44] However, a study from Nigeria found higher rate of extramarital sexual relations [29,30]. Spousal communication might help to establish harmony among family relationship and result from this study indicated low level of spousal communication about family planning. This finding is similar with previous study from Nepal based on female respondents [39]. Older age, wealthier status and education were identified common associated factors with domestic violence, and extramarital sex.

The finding revealed that older age, educated, and wealthier status of men were strongly associated with DV. However previous studies result were contradictory that young age, low education and low income were associated with DV [13-19]. Study of review on marital rape and other study from India have also contradictory results with education and DV [45]. This result is contradictory with previous research suggesting that the pressures of poverty may put a major load on families and these anxieties can explode into violence [46]. Educated male might be traditional on their attitude and practice. Nepalese societies constructed with patriarchal family, have traditional culture, and religious principles might enforce DV [26]. Similarly the finding revealed that non-indigenous people has higher probability of DV. In Nepal non-indigenous people are more devoted in religious norms than indigenous people. Systematic review of prevalence on domestic violence from different countries also highlighted that no ethnic, tribal or socio-economic group were protected by means of DV [47].

The finding indicated that educated men have less extramarital sexual relation. However previous studies from Cameroon and United States of America have contradictory findings with education status and extramarital sex [44,48,49]. Finding from this study showed that male having higher age and higher level of income was significantly associated with extramarital sex. Similar results from previous studies found that higher income and higher age were associated with extramarital sex [44,48] In contrast other literature from Bangladesh found that prominent risk of extramarital sex among lowest income groups [50]. Marital disharmony might be increased in later stage of life which is the reason behind extramarital sex among older men than younger. Most of the male reported that reasons for engaged on extramarital sex were: feeling more enjoyment with other than wife, wife complaint pain during sex after second child birth, and marital unhappiness.

Positive association were found among higher level of education, and formal employment with spousal communication but was not found protective factors. This may be due to underlying socio-religious practices among Nepalese women who are more controlled to their conservative gender roles [43]. Education consistently has been shown to influence reproductive manners. In addition, married men who had extramarital sex were less likely to had spousal communication. Systematic review from developing counties highlighted that many women still needing their husbands’ consent to use contraceptives [51]. Strong cultural, religious beliefs and conservative attitudes might be the reason for wife needed approval for contraceptive use in Nepal. Male who had conservative attitudes toward domestic violence were less likely to engage in decision making for contraceptive use. These findings are an agreement with earlier research [51]. Men who were willing to have more children were more likely to establish spousal communication. This might be the interest of men when he desires more children than he might start discussion to fulfill his desire from wife.

### Strengths and weakness of the study

The main strength was population based study that could represent from both rural and urban area. Second strength was adequate sample size for analysis, and sampling techniques for data collection. Reliability and validity were applied in each step and associated factors were set with the appropriate analytical technique.

This study provides the most comprehensive information, albeit, there are some potential limitations in the interpretation of the results for this study. First, because of the cross-sectional study design, the analysis can only offer evidence of statistical association, and cannot show cause-effect relationships. Second, in this study sample is married male, so results regarding the prevalence of domestic violence should not be generalized to all male in Nepal. Third, due to the sensitive nature of the issue, the prevalence of domestic violence and extramarital sex experience may have been underreported. However, the privacy settings adjacent the study and the excellent rapport that was built before the interview are expected to have reduced purposeful misreporting.

### Implications of findings

Study finding provides strapping evidence of eminent prevalence and increased probability of domestic violence, extramarital sex and inadequate level of spousal communication which depicts attention to key issues. Finding highlighted the need for policy maker, healthcare workforce to recognize the amplified susceptibility of both men and women with psychological problems of domestic violence, vulnerability of sexually transmitted diseases and to be arranged to recognize and tackle these concerns in policy, programs and clinical plans. Clinical and legal systems must be strong and need to develop with collaborations that can address victims’ needs and address long term or short term health impacts [52]. Qualitative and quantitative mixed method based research among both men and women need to indentify route cause of domestic violence and extramarital sex. Further intervention is needed to find out the effectively reducing vulnerability of domestic violence and extramarital sex and how to improve impact on health.

## Conclusion

The findings demonstrated that domestic violence and extramarital sex is a significant public health problem in low income countries. Males are exposed to health hazard due to their risky sexual behavior, thus sex education and awareness should be provided and promoted. Interventions focusing improved spousal communication may help prevent violence and reduce involvement on extramarital sex. Equal and immediate attention needs to be endorsed by policymakers and programmers to address these public health issues.

## Competing interests

The author declares no conflict of interest.
